# Endogenous* Fusarium* Endophthalmitis in Diabetes Mellitus

**DOI:** 10.1155/2016/6736413

**Published:** 2016-11-21

**Authors:** S. Balamurugan, Ashish Khodifad

**Affiliations:** Uvea Services, Aravind Eye Hospital, Pondicherry, India

## Abstract

Endogenous endophthalmitis accounts for 2% to 8% of cases of endophthalmitis. Immunocompromised state and intravenous drug use are the 2 most common causes of endogenous endophthalmitis due to molds fungi.* Aspergillus*,* Fusarium*, and* Scedosporium* are the common organisms in mold endophthalmitis. We report a case of* Fusarium* endophthalmitis in a patient with uncontrolled diabetes. While diabetes mellitus is a well-known risk factor for endogenous endophthalmitis, we did not find any reported case of* Fusarium* endophthalmitis in a case of diabetes mellitus. The patient presented with granulomatous uveitis masquerading as noninfectious uveitis with a very good response to steroids. The characteristic clinical features were established late in the clinical course associated with poor outcome. This case highlights the significance of uncontrolled diabetes as a risk factor for* Fusarium* endophthalmitis and also the presentation of endophthalmitis as a masquerade syndrome. The clinician should have high index of suspicion as these cases have poor outcomes.

## 1. Introduction

Endogenous endophthalmitis accounts for 2% to 8% of cases of endophthalmitis and usually occurs in a relatively immunocompromised state [1]. Predisposing conditions include diabetes mellitus, systemic malignancy, human immunodeficiency virus (HIV) infection, systemic immunomodulatory therapy and chemotherapy, intravenous drug use, organ transplantation, sickle cell anemia, and autoimmune disorders like systemic lupus erythematosus.

Endogenous fungal endophthalmitis can be considered a nonneoplastic masquerade syndrome because in many patients the condition is mistaken for noninfectious uveitis and treated with corticosteroids alone. This usually worsens the clinical course of the disease necessitating further investigation to establish the correct diagnosis. The most common agents identified as causes of endogenous endophthalmitis have been* Candida* species followed by* Aspergillus* species in various studies [2–4].

*Fusarium* is a relatively uncommon cause of endogenous fungal endophthalmitis. Endogenous* Fusarium solani *endophthalmitis has been reported in few patients with immunocompromised state [5]. We report a case of* Fusarium* endophthalmitis in a patient with uncontrolled diabetes.

## 2. Case Report

A 46-year-old Indian male presented with complaints of pain, redness, and photophobia in right eye since 2 days. He was a known case of diabetes mellitus since last 1 year and was on oral hypoglycemic agents. His past ophthalmic history was not significant. There was no history of intravenous drug use, solid organ transplant, chronic lung disease, corticosteroid treatment, and malignancy. At presentation, his BCVA was 6/6 in both eyes. On examination, his right eye showed ciliary congestion, granulomatous KPs, 2 mm hypopyon, normally reacting pupil, and early cataractous changes. His left eye had early cataractous changes. His fundus examination was normal in both eyes. Noncontact tonometry readings were 16 in right eye and 13 in left eye.

With initial clinical diagnosis of right eye acute granulomatous anterior uveitis, the differential diagnosis considered was sarcoidosis, tuberculosis, and multiple sclerosis. His ESR was 15 mm at the end of 30 minutes and 30 mm after 1 hour. He was started on topical steroids and cycloplegics. His RBS was 182 mg%. His total leucocytes counts were raised and differential count showed raised neutrophils. His Mantoux test, serum angiotensin converting enzyme, chest radiogram, and* Treponema pallidum* haemagglutination were negative. Further workup for tuberculosis was negative. ELISA for HIV was negative. With initial treatment of topical steroids and cycloplegics, his inflammation was resolving but with steroid induced IOP rise. Antiglaucoma medications were added. He was lost to follow-up.

**Figure 1:**
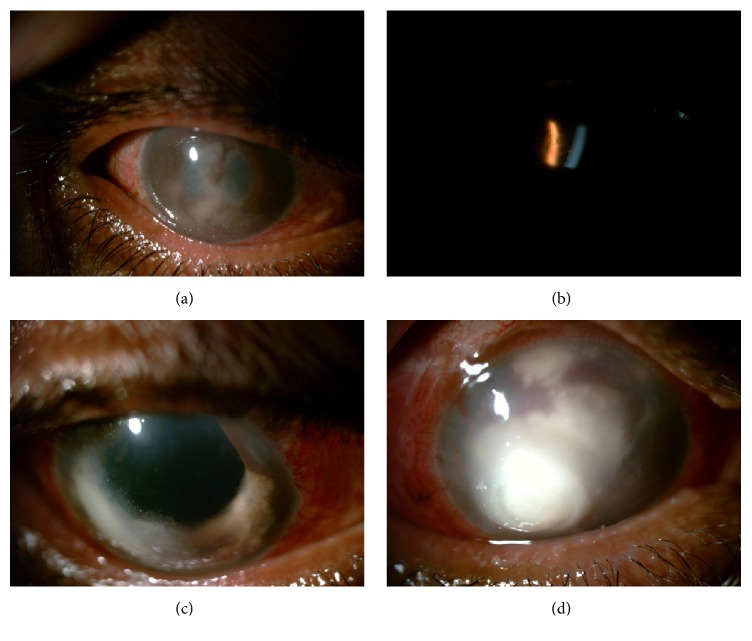
(a) Image showing ciliary congestion, granulomatous KPs, temporal iridocorneal touch, fibrinous membrane in pupillary area, 360-degree posterior synechiae, and complicated cataract. (b) Image showing presence of angle KPs. Presence of granulomatous uveitis with angle KPs and good response to steroids initially misled to the diagnosis of noninfectious etiology [sarcoidosis]. (c) Postoperative image showing corneal edema, white KPs, exudates on endothelium, and aphakia. (d) Image showing full thickness corneal infiltrate.

One month later, he presented with complaints of diminution of vision, pain, redness, and photophobia in right eye since 1 week. His BCVA was finger counting at half meter in right eye and 6/6 in left eye. His right eye showed ciliary congestion, granulomatous KPs, 1 mm hypopyon, temporal iridocorneal touch, fibrinous membrane in pupillary area, 360-degree posterior synechiae, and advanced cataractous changes (Figure 1(a)). His left eye had early cataractous changes. There was no view of fundus in his right eye due to hazy media (advanced IMC and pupillary membrane). Fundus examination was normal in left eye. His right eye B scan picture was suggestive of vitritis. noncontact tonometry readings were 16 in right eye and 15 in left eye. His RBS was 449 mg%. He was diagnosed as right eye recurrent granulomatous uveitis with secondary angle closure with complicated cataract. The differential diagnosis included sarcoidosis, tuberculosis, and endogenous endophthalmitis. In the presence of granulomatous keratic precipitates near the angles (Figure 1(b)) and prior good response to steroids, we initiated oral and topical steroids, cycloplegics, and antiglaucoma medications in consultation with physician for control of blood sugar levels. The inflammation responded to steroids and hypopyon resolved.

After control of inflammation and blood sugar levels, his right eye lens extraction was done on day 3 after presentation. On postoperative day 1, right eye BCVA was hand movement. His right eye showed corneal edema, cells 3+, flare 3+, and aphakia. His fundus was not visualized due to vitritis. Noncontact tonometry readings were 25 mm Hg in right eye and 13 mm Hg in left eye. He was continued on topical and systemic steroids, cycloplegics, antiglaucoma medications, and insulin.

On postoperative day 2, right eye BCVA was hand movement. His right eye showed corneal edema, white KPs, exudates on endothelium, hypopyon, and aphakia (Figure 1(c)). He underwent right eye AC tap and vitreous tap along with intravitreal vancomycin and ceftazidime along with intracameral vancomycin and ceftazidime (half dose). He was started on systemic antibiotics in addition to other medications. Vitreous and AC tap reports were negative for stain and culture. His postprandial blood sugar (2 hours) was 330 mg%.

Eventually, exudates and hypopyon increased with involvement of corneal stroma. Right eye core vitrectomy with intravitreal vancomycin and ceftazidime were given. Vitreous and AC tap were sent for stain and culture. Stain reports were negative. Culture reports showed* Fusarium*. Patient was started on topical and systemic antifungals. Intrastromal and intracameral voriconazole were given. Patient developed full thickness corneal infiltrate. Patient was advised right eye therapeutic keratoplasty but patient refused and he was discharged on request. On follow-up, intrastromal and intracameral voriconazole were given. On last follow-up, patient had multiple choroidal detachments with corneal infiltrate (Figure 1(d)).

## 3. Discussion

Immunocompromised state and intravenous drug use are the 2 most common causes of endogenous endophthalmitis due to molds [6].* Aspergillus*,* Fusarium,* and* Scedosporium* are the common organisms in mold endophthalmitis. The review of literature by Malavade et al. [5] shows that* Fusarium* endophthalmitis is most common in patients with hematological malignancies. While diabetes mellitus is a well-known risk factor for endogenous endophthalmitis, we did not find any reported case of* Fusarium* endophthalmitis in a case of diabetes mellitus.

In this case, the causative organism as detected by culture is* Fusarium*. The source of infection can be exogenous or endogenous. The absence of any trauma or surgery before onset of disease rules out exogenous invasion by the fungus. The mechanism of infection is probably endogenous. The presence of uncontrolled diabetes and gradual evolution of uveitis to eventually involve the other structures point to the probable endogenous mechanism. The systemic focus/source of infection could not be identified as patient was lost to follow-up.

In contrast to* Fusarium* endophthalmitis, the endophthalmitis caused by aspergillus was most commonly found in intravenous drug use (27%), solid organ transplant (23%), chronic lung disease (17%), corticosteroid treatment (43%), hematologic malignancy (8%), and other malignancy (1%), as noted by Riddell IV et al. [7] in review of literature of endogenous* Aspergillus* endophthalmitis from 1949 to 2001.

Endogenous fungal endophthalmitis develops slowly as multifocal areas of chorioretinitis with vitritis, hypopyon, granulomatous, or nongranulomatous inflammation with keratic precipitates. The signs pointing to fungal etiology include corneal infiltrates or edema, immobile hypopyon, mass in iris or ciliary body, necrotizing scleritis, and string of pearls in vitreous cavity. In this case, all of these classical signs did not present till late stages. Endogenous fungal endophthalmitis is known to masquerade as noninfectious etiologies and often responds to steroids in initial stages. In this case also, the initial clinical picture, B scan picture, and initial response to steroids were misleading clues to noninfectious etiology.

The endogenous* Fusarium *endophthalmitis in this eye is characterized by massive ingrowth of the fungi in all areas containing basement membrane collagen, that is, Descemet's membrane, lens capsule, and internal limiting membrane of the retina. Based on the clinical features and in vitro studies, Jørgensen and colleagues [8] speculated that* Fusarium solani *has an affinity for basement membrane collagen and integrin and therefore the ingrowth of the fungi is most pronounced in these areas. Similar clinical findings were noted in our case also. There was a presence of a biofilm over the anterior lens capsule and the involvement of Descemet's membrane.

The endophthalmitis was not postoperative fungal endophthalmitis because, postoperative fungal endophthalmitis most commonly presents after 4 weeks and in rare cases can present as early as 5 to 7 days. In our case, the findings were already present preoperatively and the classic fungal picture developed in first 2 postoperative days.

The visual outcome of* Fusarium* endophthalmitis, as reported in the literature, is poor [5]. Despite all the attempts, similar outcome was noted in our case also. As observed in some of the reported cases in literature, the septic focus could not be identified in our case also. As the patient was lost to follow-up, the systemic outcome could not be noted.

## 4. Conclusion

To the best of our knowledge, this is the first case of* Fusarium* endophthalmitis reported in a diabetic patient. This case highlights the significance of uncontrolled diabetes as a risk factor for* Fusarium* endophthalmitis and also the presentation of endophthalmitis as a masquerade syndrome. The clinician should have high index of suspicion as these cases have poor outcomes.
